# Gene Expression Analysis before and after the Pelvic Flexure in the Epithelium of the Equine Hindgut

**DOI:** 10.3390/ani14162303

**Published:** 2024-08-08

**Authors:** Cameron D. Moss, Amber L. Wilson, Kailee J. Reed, Kaysie J. Jennings, Isabelle G. Z. Kunz, Gabriele A. Landolt, Jessica Metcalf, Terry E. Engle, Stephen J. Coleman

**Affiliations:** 1 Department of Animal Sciences, Colorado State University, Fort Collins, CO 8023, USA; cameron.moss@colostate.edu (C.D.M.); isabelle.gz.kunz@gmail.com (I.G.Z.K.); terry.engle@colostate.edu (T.E.E.); 2 Watchmaker Genomics, Boulder, CO 80301, USA; 3 Transnetyx, Memphis, TN 38016, USA; 4 Department of Clinical Sciences, Colorado State University, Fort Collins, CO 8023, USA

**Keywords:** pelvic flexure, equine, gastrointestinal, homeostasis, hindgut, microbiome, RNA sequencing

## Abstract

**Simple Summary:**

The equine hindgut plays a central role in digesting forage by hosting a complex community of microorganisms responsible for fermenting and digesting plant fibers. Research has shown that the different compartments of the hindgut have unique microbial communities. The pelvic flexure, an anatomic structure that separates segments of the hindgut and prevents backflow, seems to help maintain these distinct communities. How it achieves this has yet to be fully understood. While much is known about the hindgut’s anatomy and digestion, only a few studies have investigated gene expression patterns and differences in the tissues around the pelvic flexure. In this study, we used next-generation RNA sequencing to analyze and compare gene expression in the pelvic flexure and the ventral and dorsal segments of the large colon in horses. This knowledge can help researchers, veterinarians, and horse owners better understand the equine hindgut’s physiology and how horses interact with the microbial communities there and could provide insights for managing healthy horses or treating horses with digestive issues.

**Abstract:**

Previous research demonstrated the distribution of distinct microbial communities in the equine hindgut surrounding the pelvic flexure. The current study evaluated gene expression in epithelial tissues surrounding the pelvic flexure to characterize patterns that might correlate with microbial distribution. Gene expression was determined by analyzing RNA sequence data from the pelvic flexure, the left and right ventral colon, and the left and right dorsal colon. An average of 18,330 genes were expressed across the five tissues sampled. Most of the genes showed some level of expression in all five tissues. Tissue-restricted patterns of expression were also observed. Genes with restricted expression in the left ventral and left dorsal colons have communication, signaling, and regulatory functions that correlate with their known physiology. In contrast, genes expressed exclusively in the pelvic flexure have diverse functions. The ontology of genes differentially expressed between the pelvic flexure and the surrounding tissues was associated with immune functions and signaling processes. Despite being non-significant, these enrichment trends were reinforced by the functions of statistically significant expression differences between tissues of the hindgut. These results provide insight into the physiology of the equine hindgut epithelium that might influence the microbiota and its distribution.

## 1. Introduction

The equine gastrointestinal (GI) tract, a crucial and complex organ system, is divided into the foregut and hindgut. The hindgut, consisting of the cecum, large colon, and small colon, is the primary site for fiber digestion and water resorption in the equine GI tract [1,2]. As hindgut fermenters, horses derive over 60% of their dietary energy from the digestion of plant-based fibers like cellulose in the cecum and ventral colon [3]. The equine hindgut’s microbiota, a key player in digestion, supports microbial fermentation that produces volatile fatty acids (VFAs), a primary energy source crucial to equine digestive physiology. Disruptions in the hindgut microbiota can lead to performance deficits, health issues, and mortality [4,5,6,7,8,9].

Homeostasis is a self-regulating process in biological systems that maintains stability while allowing the system to respond to changing external conditions [10]. Maintaining homeostasis is essential for any living system to operate efficiently and effectively [11,12]. GI homeostasis is influenced by the constant interactions of the host’s tissues and physiology, the microbiota, nutrients, and other factors [13,14]. The structure and function of the various hindgut compartments of the equine hindgut are critical for digestive physiology, microbiota support, GI health, and homeostasis [15,16,17]. Previous work by our group demonstrated that the pelvic flexure, a structural element, plays a significant role in defining distinct microbial communities (cecum and ventral colon from dorsal colon and feces) of the equine hindgut, enlightening us about this crucial aspect of equine anatomy [18]. The pelvic flexure, a horseshoe-shaped bend between the ventral and dorsal colons, helps to define the hindgut compartments and prevents the backflow of digesta. It is important to note that the pelvic flexure is not a physical barrier and is not supposed to block transit through the GI tract. It can often be the site of obstruction, resulting in colic [19]. The physiology of equine digestion [20,21,22,23] and the anatomy of the hindgut [19] have been well reviewed. There have been relatively few studies of gene expression or differential patterns in the tissues of the equine hindgut to complement our understanding of digestive physiology. It is not yet known how gene expression in tissues of the hindgut impacts digestive physiology and mediates interactions with the microbiota. Furthermore, it remains unclear how disruptions can result in disease pathology and dysfunction. A crucial first step in developing this understanding is to investigate gene expression in the tissues of the GI tract from healthy animals to establish a baseline pattern of expression across the various compartments of the hindgut.

In the present study, we used RNA sequencing to investigate gene expression patterns in the intestinal epithelium of the pelvic flexure and surrounding ventral and dorsal colons. These regions were selected for this analysis as they correspond anatomically with the distinct microbial communities of the equine hindgut [18]. The objective was to determine baseline expression profiles in the equine GI to better understand intestinal epithelial cell function related to hindgut digestive physiology and identify gene expression differences between the hindgut compartments, which may contribute to the distinct microbial communities observed in each.

## 2. Materials and Methods

### 2.1. Animal Subjects and Sample Collection

Samples were collected from three four-year-old quarter horses (two males and one female) randomly selected from a larger group for an unrelated project. The Colorado State University Institutional Animal Care and Use Committee approved animal care, handling, and euthanasia (protocol 16-6405A). The horses were euthanized for reasons unrelated to gastrointestinal disease. All three horses had body condition scores (BCS) between 5 and 6 [24], were free of any diagnosable gastrointestinal issues, did not receive prior antibiotics or non-steroidal anti-inflammatory drugs, and were fed mixed grass hay with ad libitum access to food and water.

Intestinal epithelial samples were collected from the right ventral colon (RVC), left ventral colon (LVC), pelvic flexure (PF), left dorsal colon (LDC), and right dorsal colon (RDC) between 40- and 45-min post-mortem. The GI tract was removed, and each compartment was identified, beginning with the pelvic flexure. The left ventral and left dorsal colon were collected 10–15 cm caudal or rostral from the PF. The right ventral and right dorsal colon samples were collected from the opposite end of each compartment relative to the PF. A 2 to 3 cm^3^ full-thickness section was cut from each site with a sterile scalpel and transferred to a clean sample cup. The tissue was rinsed with sterile PBS before dissection of the mucosal and submucosal layers from the serosal muscle. The mucosal/submucosal epithelium was divided into approximately 0.5 cm^3^ pieces and placed in 5 mL of RNALater^TM^ (Thermo Fisher Scientific, Waltham, MA, USA). The tissues were incubated at 4 °C for 24 h, removed, and transferred to −80 °C for storage according to the manufacturer’s protocol.

### 2.2. RNA Isolation and Quality Control 

Total RNA isolation was performed using a modified TRIzol^TM^ (Thermo Fisher Scientific, Waltham, MA, USA) protocol [25,26]. The procedure was as follows: samples were removed from −80 °C and placed on ice to thaw; 50 milligrams of tissue was weighed into a sterile tube filled with garnet shards and a zirconium bead (D1033-30G, Benchmark Scientific Inc., Sayreville, NJ, USA), 1 ML of TRIzol^TM^ was added, and samples were homogenized using a BeadBug 3 homogenizer (Benchmark Scientific Inc., Sayreville, NJ, USA). Following homogenization, the samples were incubated at room temperature for 5 min, combined with 200 μL of chloroform, vortexed, and centrifuged to separate the aqueous and organic phases. The aqueous phase was removed to a clean tube, and the total RNA precipitated with isopropanol. All samples were treated with DNase (TurboDNase AM2238, Thermo Fisher Scientific, Waltham, MA) to remove genomic DNA contamination. The quality of the isolated samples was verified by checking concentration and purity on a NanoDrop 1C (Thermo Fisher Scientific, Waltham, MA, USA) and integrity using a QuBit 4 (Thermo Fisher Scientific, Waltham, MA, USA) and the RNA IQ assay (Thermo Fisher Scientific, Waltham, MA, USA). All samples had concentrations above 200 ng/μL, A260/280 and A260/230 ratios above 1.7, and RNA IQ scores of at least 7.

### 2.3. Library Prep and Sequencing

mRNA sequencing libraries were prepared for each tissue sample along with a “no-RNA” (water) negative control using the NEBNext^®^ Ultra II Directional RNA Library Prep Kit for Illumina (Catalog # 7760S, New England Biolabs Inc., Ipswich, MA, USA). Messenger RNA was isolated from total RNA using the NEBNext^®^ Poly(A) mRNA Magnetic Isolation Module (Catalog # E7490, New England Biolabs Inc., Ipswich, MA, USA) and fragmented with a target size of 200 nucleotides. First- and second-strand synthesis proceeded according to the manufacturer’s protocol. Each of the 15 samples and the negative control were indexed using a unique oligo sequence from the NEBNext^®^ Multiplex Oligos for Illumina Sets 1 and 2 (Catalog # E7335S and E7500S, New England Biolabs Inc., Ipswich, MA, USA). Following PCR enrichment, library size, quality, and abundance were assessed using an Agilent 2200 Tapestation (Agilent Technologies Inc., Santa Clara, CA, USA) and High-Sensitivity D1000 Screen Tape (Catalog # 5067-5584, Agilent Technologies Inc., Santa Clara, CA, USA). Equimolar quantities of each library were combined, and the size, quality, and abundance of the combined libraries were re-assessed. Sequencing was performed on an Illumina NextSeq 500 (Illumina Inc., San Diego, CA, USA) using a NextSeq 500/550 75-cycle v2.5 High-Output kit (Catalog # 20024906, Illumina Inc., San Diego, CA, USA).

### 2.4. Bioinformatic Analysis

**Figure 1:**
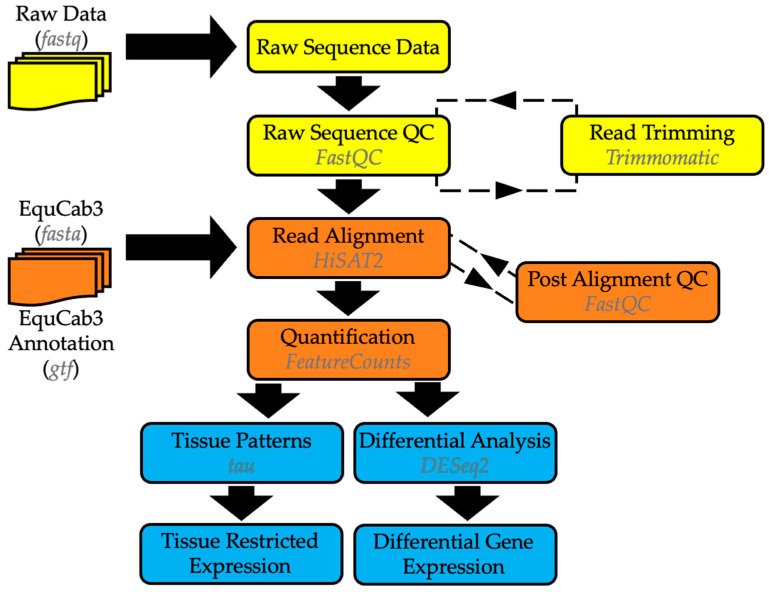
Gene expression analysis workflow. The steps highlighted in yellow involve preparing the RNA-seq data for analysis. Steps in orange detail mapping the data to the reference genome and annotation, while the steps in blue describe the gene expression prior to functional analysis.

Data from the sequencer were uploaded to BaseSpace^TM^ (Illumina Inc., San Diego, CA, USA) and demultiplexed to generate individual FASTQ files for each tissue sample (n = 3 per tissue). A diagram of the analysis workflow is shown below in Figure 1. The Galaxy platform [27] and the CU/CSU Summit high-performance computing system were used to assess read quality via FastQC [28] and MultiQC [29]. Read trimming was achieved via Trimmomatic [30], and read alignment to EquCab3 used HISAT2 [31]. Post-alignment QC was also performed using FastQC. Gene quantification used the equine gene annotation from Ensembl 106 [32] and was performed using featurecounts [33]. Genes were detected in a tissue with at least one read assigned and labeled as “expressed” in a tissue if they had detectable expression in at least two of the three horses. Differential gene expression was analyzed using DESeq2 [34], and a Benjamini–Hochberg correction was applied to account for multiple testing. The comparisons used were (1) RVC versus PF, (2) LVC versus PF, (3) LVC versus LDC, (4) LDC versus PF, and (5) RDC versus PF. Tissue specificity was assessed using the list of normalized expression values generated by DESeq2. A tau index value was calculated for each gene across all tissues. Tau index analysis was performed using the Tau algorithm protocol [35,36,37]. This analysis ranked individual genes from 0 to 1, with a tau value of 0 indicating consistent expression across all five tissue sites and 1 indicating tissue-restricted expression.

Functional analysis of the differentially expressed genes identified between the five tissue sites was performed using the functional annotation tool DAVID [38,39]. Biological processes, molecular function, and cellular components were all included in the analysis. The input lists used for the analysis were those of differentially expressed genes identified and described above using DESeq2 against a background of all annotated genes from the Ensembl 106 annotation. Enrichment analysis was performed to determine if gene ontology terms were overrepresented in the differentially expressed gene lists.

## 3. Results

### 3.1. Sequencing Results

**Figure 2:**
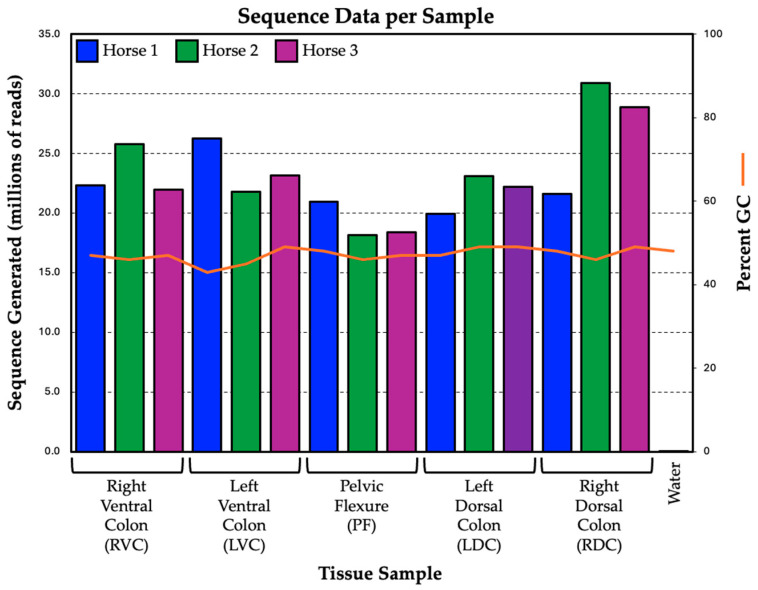
Summary of sequence data generated by sample. The left Y-axis indicates the total reads generated, and the right Y-axis indicates the percent GC content (orange line). Data are grouped by tissue along the X-axis and horse (blue, green, and purple columns; see chart legend for associations).

Sequencing of 16 samples (15 tissue samples and the negative control) generated a total of 360,021,067 76-basepair sequence reads. Sequence data are available in the Sequence Read Archive under Bioproject PRJNA631014. A total of 14,468,231 reads were removed from the analysis during the demultiplexing step as they could not be confidently assigned to a sample group based on the index sequence. The average sequence generated for the fifteen tissue samples was 23,033,024 reads with a range of 18,178,616 (pelvic flexure—Horse 2) to 30,913,609 (right dorsal colon—Horse 2) reads. In contrast, the negative control produced only 57,475 (0.016% of total) sequence reads. While assessing the sequence quality, it was observed that the first 8–10 base pairs of the reads in each sample had lower per-base quality and an unexpected distribution of the per-base sequence content compared to the other 66 bases of the read. They were removed to avoid any potential ambiguity resulting from including these bases in downstream analyses. The average GC content of all samples was 47.1%, with a range of 44% to 49%. Figure 2 displays the total sequence generated and %GC content for the fifteen tissue samples compared with the negative control. Supplementary Table S1 shows the sequencing results for all samples.

### 3.2. Mapping Results

Sequence reads assigned to the individual tissue samples were aligned to the equine reference genome (EquCab3) using HISAT2. The average overall alignment rate (unique and multiple mapping reads) was 93.44%, ranging from 89.16% to 95.89%. A complete mapping summary is presented in Supplementary Table S2.

### 3.3. Gene Expression

**Figure 3:**
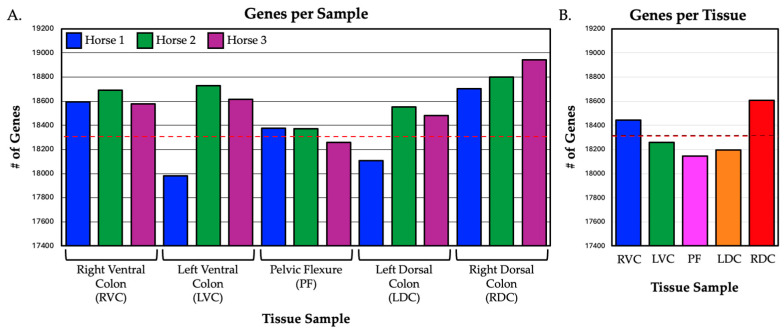
The number of genes expressed in sections of the equine hindgut by (**A**) sample and (**B**) tissue. The average across all tissues was 18,330 +/− 191 genes (red dashed line).

Raw read count data were summarized by sample using the featureCounts program and the Ensembl 106 annotation release of the equine reference genome. Normalized count values were produced using DESeq2. The number of genes expressed in each tissue (reads mapped to annotated gene model in at least 2 of 3 horse samples for that tissue) was 18,445 in the RVC, 18,258 in the LVC, 18,146 in the PF, 18,195 in the LDC, and 18,606 in the RDC, respectively. The number of annotated genes with expression was also similar across subjects and sampling sites, with an average of 18,330 +/− 191 and a range of 18,146–18,606. These results indicate that the data is consistent, comparable, and free of issues that might impair the subsequent analyses. The number of expressed genes by sample and summarized by tissues is displayed in Figure 3.

**Figure 4:**
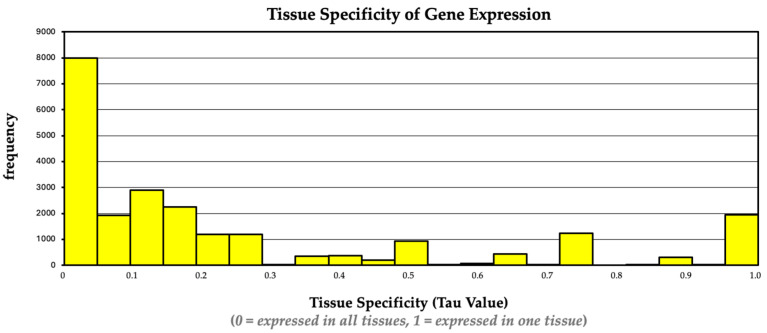
Tau index value frequency distribution across hindgut locations. Tau index values ranged from 0 to 1, with 0 indicating consistent expression across hindgut tissues and 1 indicating differential expression (i.e., tissue-restricted expression in 1 of the five hindgut areas). The frequency displays how many genes were categorized at a particular tau index value.

Patterns of expression were compared across the five sampling locations. Of the 31,215 gene features included in the Ensembl 106 annotation, expression was detected for a majority, 16,750 (53.7%), at all five intestinal sites. Tissue-specificity was determined by calculating a tau index value. The tau index value frequency distribution across the five tissues is presented in Figure 4. All unique, tissue-restricted genes were filtered based on whether they were expressed in 2 or more horses and if that expression was exclusive to 1 of the five tissue areas. This resulted in a total of 1203 genes assessed as being tissue-restricted in this dataset. The top 20 unique genes per tissue site are shown in Table 1. Tissue-restricted gene expression was found in all five tissue sites, with the pelvic flexure having the least (179 genes) and the right dorsal colon having the most (311 genes). A complete list of genes with a restricted expression pattern supported by data in at least two horses is included in Supplemental Table S3.

### 3.4. Differential Gene Expression

**Figure 5:**
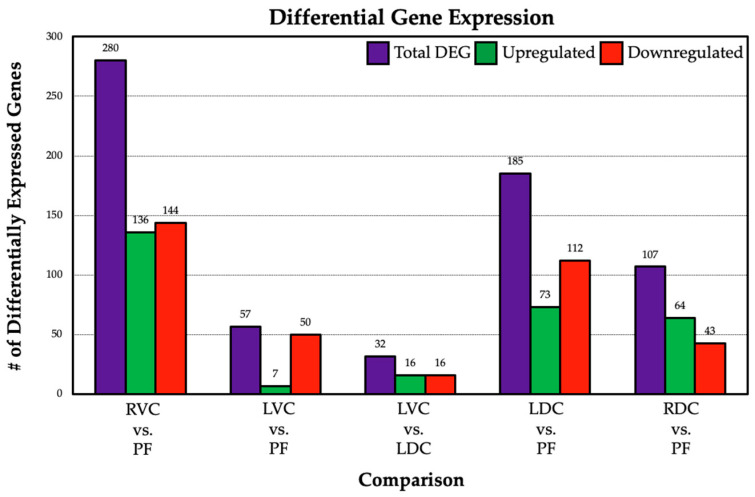
Differential expression by tissue comparison. Purple indicates the total number of differentially expressed genes, green indicates a higher abundance in the first tissue of the comparison, and red indicates a higher abundance in the second tissue.

Differential gene expression between the GI regions was determined using DESeq2. The specific comparisons identified the following numbers of significantly differentially expressed genes: (1) PF versus RVC = 280, (2) PF versus LVC = 57, (3) LVC versus LDC = 32, (4) PF versus LDC = 185, and (5) PF versus RDC = 107. The number of differentially expressed genes, including those that were up- and down-regulated, is presented in Figure 5.

**Figure 6:**
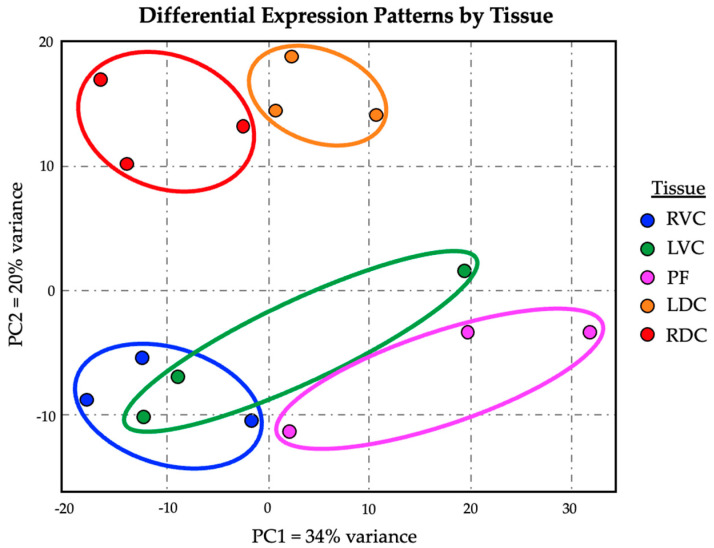
Principal component analysis showing expression differences between the right and left ventral colon (RVC and LVC), the pelvic flexure (PF), and the left and right dorsal colon (LDC and RDC).

Lists of the differentially expressed genes (Benjamini–Hochberg-adjusted *p*-value < 0.05) from each comparison are presented in Supplemental Tables S4–S8. A principal component analysis (or PCA) was generated using the differential expression data generated by DESeq2. The graph resulting from the first two principal components (PC1 and PC2) is presented in Figure 6. PC1 accounts for 34% of the variance in differential gene expression and appears to correlate with anatomical location along the hindgut. The LVC and LDC are closer anatomically to the pelvic flexure and closer on the graph than the RVC and RDC, which are more distal anatomically. PC2 accounts for 20% of the variance and appears to differentiate the ventral and dorsal colons.

### 3.5. Functional Analysis

**Figure 7:**
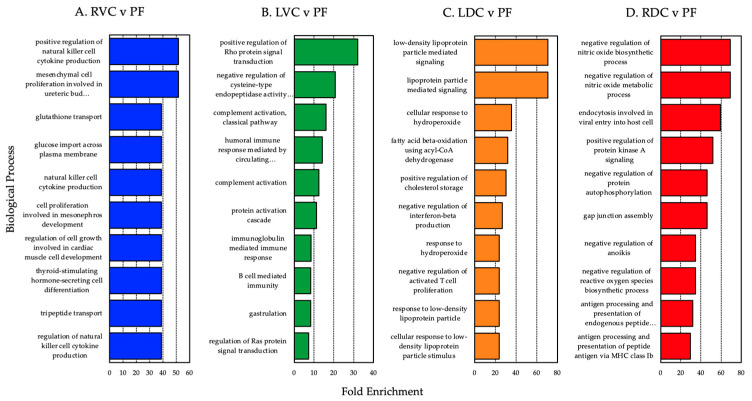
Top 10 biological processes enriched in the comparisons of differential expression between the right ventral colon, left ventral colon, left dorsal colon, right dorsal colon, and pelvic flexure.

Enriched gene ontology terms in the lists of differentially expressed genes were determined using DAVID. The top 10 biological processes enriched in each tissue comparison for differential expression are displayed in Figure 7. The entire list of enriched GO categories for each comparison is included in Supplemental Tables S9–S13.

## 4. Discussion

The equine hindgut hosts diverse microbial communities critical for digesting a horse’s forage-based diet. The pelvic flexure—a common site of impactions, colic, and other digestive disorders [19]—helps regulate transit in the hindgut and, it would seem, segregates distinct microbiota [18]. The roles of the various hindgut segments (cecum, ventral colon, dorsal colon, small colon) in digestive and absorptive processes have been well studied [19,20,21,22,23]. Physiological differences between the GI regions at the gene expression level have not been determined. The present study used RNA sequencing to investigate differential gene expression in the intestinal epithelium of the equine hindgut, specifically in the regions surrounding the pelvic flexure. Establishing and maintaining homeostasis in the hindgut is critical for normal physiology and function. The tissues sampled for this study were all the mucosal and submucosal epithelial layers of the hindgut in the ventral colon, pelvic flexure, and dorsal colon. Given the broad similarities observed in structure and function based on the shared type and distribution of cells in these intestinal epithelia, we anticipated that gene expression patterns at all five tissue sites sampled would be broadly similar. Still, given that the functional roles of the ventral and dorsal colons differ in equine digestive physiology, we also expected to find both tissue-restricted and different gene expression profiles across the five locations. The results of the current study provide new insight into the gene expression patterns underlying intestinal physiology in various sections of the equine hindgut. They could inform understanding of interactions with the intestinal microbiota.

Most genes expressed in this dataset (16,750/31,215 or 53.7%) had detectable expression at all five sample locations (i.e., at all 5 GI sites). It is important to note that just because a gene was identified in all five tissue locations does not indicate that the expression level detected at all five sites was equivalent. Broadly, the functional roles associated with this gene category were related to cellular metabolism, catalytic activity, cellular responses to stimuli or stress, and other shared components of physiology.

In addition to the genes with detectable expression at all five sample sites, 1203 genes were identified in only 1/5 of tissues, indicating tissue-restricted expression patterns within this dataset. These tissue-restricted may be informative about the unique physiology of the different tissue sites. They may also help explain the diverse microbiota hosted in the different hindgut segments. However, that connection will need to be addressed in future work.

The ventral colon of the equine hindgut extends from the cecum to the pelvic flexure. Its primary roles in digestion include support of microbial fermentation of dietary fiber and some absorption of the products resulting from that fermentation. Genes with expression restricted to the LVC include forkhead box B1 (FOXB1) and insulin receptor substrate 4 (IRS4). FOXB1 is a member of the forkhead box family of transcription factors. These transcription factors are involved in various biological processes, including cell proliferation, cell differentiation, immune responses, and signaling. Several related forkhead box family members (FOXA1, FOXA2, and FOXO1) have been implicated in epithelial cell development and commensalism [40,41,42], indicating the potential for FOXB1 to be involved in similar processes. IRS4 expression has been associated with cell proliferation, and its overexpression has been correlated with the development and staging of colorectal cancers [43]. IRS4 has been poorly studied but may be involved in regulating cell proliferation in the ventral colon. Another gene with expression restricted to the LVC was cadherin 8 (CDH8), an integral membrane protein associated with calcium-dependent cell-to-cell adhesion. The expression of CDH8 in the LVC could help ensure proper cell-to-cell interactions to help maintain the integrity of the mucosa in that hindgut region [44]. A final example of a gene with expression restricted to the LVC in our dataset was the cholecystokinin B receptor (CCKBR) gene. CCKBR encodes a G-protein coupled receptor for gastrin and CCK. It has demonstrated expression in the brain and gastrointestinal tract, primarily in the stomach, enhancing mucosal growth [45,46]. CCKBR has also been associated with biological processes involved in pH regulation, processes which may be necessary as digesta transition from the ventral to dorsal colons.

The dorsal colon extends from the pelvic flexure to the transverse colon and runs along the dorsal aspect of the horse’s intestinal cavity. The role of the dorsal colon is the absorption of water, electrolytes, and volatile fatty acids (VFAs), which result from microbial fermentation in the cecum and ventral colon. As with the LVC, several genes appeared to have expression restricted to the LDC. Solute carrier family 24 member 1 (SLC24A1) encodes a potassium-dependent sodium/calcium exchanger protein family member [47]. Notably, this gene has relatively broad expression across multiple tissue types but was only detected in the LDC from our samples. SLC24A1 has been implicated in maintaining intestinal homeostasis by promoting ion balance/transport, absorption/secretion of molecules in the intestinal lumen, or late-stage nutrient absorption in the small intestine [48]. As the dorsal colon is a primary site of absorption, the expression of SLC24A1 in the LDC may be associated with that task. Finally, SLC24A1′s functional role in ion transport and membrane potential is essential in maintaining GI homeostasis in this region of the hindgut, in particular as it relates to the proper absorption of nutrients at the proximal dorsal colon region that may directly influence absorption processes in the RDC. Another gene with LDC-restricted expression in our data was cytochrome P450 subfamily A member 2 (CYP1A2), which codes for a member of the cytochrome P450 superfamily. This family of enzymes is involved in drug metabolism and cholesterol, steroid, and lipid synthesis [49,50], with demonstrated interactions of these functions and the intestinal microbiota [51,52]. There is not much information regarding the expression of this enzyme superfamily in the hindgut. Still, the expression of CYP1A2 in the LDC may be associated with biological processes involved in metabolizing microbial fermentation’s absorbed products. Finally, our data shows that transglutaminase 5 (TGM5) expression appears restricted to the LDC. Transglutaminases can stabilize protein structures by catalyzing glutamine-lysine crosslinking. This stabilization can improve barrier function in the epidermal layers of the skin [53,54]. In the LDC, it could be essential to help maintain mucosal integrity.

Our dataset showed several genes of interest with expression restricted to the pelvic flexure region of the hindgut. First was the steroid receptor-associated and regulated protein (SRARP) gene. SRARP is currently described as enabling estrogen receptor binding and positive estrogen receptor signaling pathways regulation. The presence of estrogen receptors in IECs has been previously reported [55], and estrogen signaling has been implicated in the modulation of epithelial cell secretion and epithelial barrier functions [56,57]. There is also evidence that glucocorticoid hormones in the intestinal epithelium help regulate T-cell activation [58]. Together, these functions may provide insight into how distinct microbial communities are separated at the pelvic flexure. However, additional investigation is necessary.

Similarly, expression of the transketolase-like 1 (TKTL1) gene was also found to be restricted to the pelvic flexure. The associated protein forms a homodimer complex to convert intermediate metabolites and links the pentose phosphate and glycolytic pathways. The expression of transketolase supports ATP production, which would be required in metabolically active tissues [59]. In IECs, TKTL1 expression has also been demonstrated to help maintain epithelial barrier function and inhibit apoptosis-induced colitis [59]. A final gene with expression restricted to the pelvic flexure in our data was neurotrimin (or NTM), a protein-coding gene that promotes neurite outgrowth and adhesion, stabilizes synapses, and is closely linked to a related opioid-binding protein/cell adhesion molecule-like (OPCML) family member [60,61,62]. As a possible explanation for its expression in the pelvic flexure, this could indicate the importance of proper enteric innervation, structural sensing, stabilization, and community interaction with other cells and contents of the intestinal lumen in this region specifically. Such interactions may be possible at the pelvic flexure because of its narrower luminal space. This is especially true compared to the broader structures of the equine ventral and dorsal colons. Additional research is required to determine whether this explanation is possible.

As discussed, all five sites in the equine hindgut region sampled for this study had a broad distribution of gene expression. On average, 18,330 +/− 191 genes had detectable expression in each tissue, representing 58.7% of the features in the Ensembl 106 equine annotation set. Further, 16,750 annotated genes had detectable expression in all five tissue locations. Overall, the differences in the expression patterns of the tissues were relatively narrow, with the number of differentially expressed genes ranging from just 32 (left ventral versus left dorsal colons) to 280 (pelvic flexure versus right ventral colon). It is important to remember that all samples used in this analysis comprised intestinal epithelial cells collected from the mucosal layer of the equine large colon. They are, therefore, compositionally comparable and so logically would be expected to share broadly similar expression patterns.

There are also important structural and physiological differences between the ventral colon, dorsal colon, and pelvic flexure to appreciate. The ventral colon has a uniform diameter over its length, and the luminal space is surrounded by four bands of smooth muscle on either side. In contrast, the pelvic flexure has a relatively narrow intestinal structure, lacks sacculation, and only has a single band of muscle. The diameter expands towards the distal end of the dorsal colon and incorporates additional bands of muscle and subtle sacculations [2]. Physiologically, the ventral colon supports many essential digestive and absorptive functions—not the least of which is microbial fermentation—while the dorsal colon is mainly responsible for the absorption and transportation of ingesta, including the last-minute absorption of electrolytes, solutes, and water. The principal component analysis completed using the DESeq2 analysis highlights the gene expression differences associated with these structural and functional distinctions. The expression patterns of the five tissue sites appear to separate based on location within each intestinal compartment (principal component 1) and by the intestinal compartments themselves (principal component 2). The ventral and dorsal colons, including the left and right aspects of each one, are well distinguished based on observed differences in gene expression. The pelvic flexure is distinct from the ventral and dorsal colons but appears to cluster with the ventral colon. The basis of this separation is further supported by identifying highly variable genes within the dataset. As has been presented as the motivation for the current study, previous work by our group [18] highlighted and supported the existence of distinct microbial communities in the equine hindgut separated by the pelvic flexure. The fact that the ventral and dorsal colons are distinguished by the microbial communities they support and their gene expression profiles add credence to the possibility that gastrointestinal and digestive physiology aspects play a role in influencing that microbiota. Additional work will be required to determine if and how exactly host physiology could impact the composition of the gastrointestinal microbial communities. The expression differences identified by our study provide new avenues for future investigations.

Gene ontology terms associated with the lists of differentially expressed genes identified by DESeq2 were evaluated for over-representation of functional categories or descriptors. The relatively small size of the data set (three biological replicates) and narrow lists of differentially expressed genes identified between sampling sites limited the effectiveness of this analysis. Multiple testing corrections eliminated all statistical significance. Still, there were some potentially valuable trends in the results, which may help and direct future investigation. Each differentially expressed gene list appeared enriched with biological processes associated with adaptive immune responses (both humoral and cell-based) (Figure 7).

Based on our analyses, there appears to be an apparent shift from humoral to cell-based immune responses moving from the proximal (right ventral colon) to the distal (right dorsal colon) ends of the large colon. This transition is highlighted by comparing the categories represented before and after the pelvic flexure. In the ventral colon, the top categories reflect humoral and immunoglobulin-mediated immune responses (GO:0006959, GO:0002455, GO:0016064), B-cell mediated immunity (GO:0019724), and complement activation (GO:0006956, GO:0006958). In the dorsal colon, several of the top categories relate to the regulation of T-cell proliferation (GO:0046007), antigen processing and presentation via MHC class II (GO:0002504), and the regulation of interferon-beta production (GO:0032688). Additionally, multiple ontologies are associated with lipoprotein-particle signaling and responses (GO:0055096, GO:0010886, GO:0055098, GO:0071404, GO:0034372). The appearance of these biological processes following the pelvic flexure likely reflects the dorsal colon’s role in the absorption of nutrients resulting from the microbial fermentation and digestion occurring in the ventral colon but are also linked with inflammation and immune responses, which may also contribute to the immune system’s role in influencing the distribution of various microbial constituents in the different compartments of the GI tract. Further investigation is necessary to generate additional data confirming these results and analyze the specific genes expressed in this case.

Our previous work focused on the pelvic flexure as an important anatomical marker that separated distinct microbial communities in the equine hindgut [18]. Our goal in this work was to investigate the gene expression underlying physiological differences in the hindgut regions surrounding the pelvic flexure. As such, we highlighted the patterns of differential expression for genes with distinct expression in the pelvic flexure and demonstrated a difference relative to the other regions (ventral and dorsal colon), the upstream region (ventral colon), or the downstream region (dorsal colon).

Several genes with notable annotated functions were more abundant in the pelvic flexure than the ventral and dorsal colons. The ABO alpha 1-3-N-acetylgalactosaminyltransferase and alpha 1-3-galactosyltransferase (ABO) and SHANK-associated RH domain interactor (SHARPIN) genes were both detected at higher levels in the PF compared to the RVC, LDC, and RDC. In humans, the ABO gene indirectly encodes the ABO blood group antigens, with the A and B alleles each encoding a glycosyltransferase that actively catalyzes the final step in synthesizing the A and B antigens. These antigens are expressed on red blood cells, in tissues of the salivary glands, and—notably—on epithelial cells in the GI tract [63]. It has also been demonstrated that individuals with different ABO antigens (A, B, AB, or O) present with distinct populations and diversity metrics for the bacterial populations in their intestinal microbiota [64]. Horses do not have the ABO blood groups that are seen in humans and other species, but its apparent expression and increased abundance of ABO in the pelvic flexure compared to the ventral and dorsal colons could be associated with the separation of distinct microbial communities reported by Reed et al. [18]. SHARPIN is a highly conserved autosomal gene that is a part of the linear ubiquitin chain assembly complex (LUBAC) and plays a key role in regulating immune and inflammatory responses by enabling polyubiquitin modification-dependent protein-binding activity and being actively involved in protein linear polyubiquitination and signal transduction regulation [65,66,67,68]. Linear poly-ubiquitin chains are widely involved in innate and adaptive immune signaling pathways [6]. SHARPIN has been found to help initiate systemic inflammatory responses and regulate cell survival and apoptosis. It is an essential regulator of immune and inflammatory responses [65,69]. It is, therefore, plausible that SHARPIN’s higher expression exclusively in the PF directs specific immune-related responses that could contribute to the microbial differences observed before and after the PF.

Another notable gene, microsomal glutathione S-transferase 1 (MGST1), was detected at higher levels in the PF versus the LVC, LDC, and RDC. MGST1 codes for a protein that is an important mediator of inflammation and plays an essential role in pathways associated with the innate immune system. Upregulation of MGST1 has been implicated in initiating changes related to oxidative stress resulting from inflammatory bowel disease (IBD) pathogenesis in rat models. In these experiments, IBD altered the epithelial expression of MGST1, resulting in differing metabolite profiles and changes to the colonic microbiome [70]. In humans with Crohn’s disease or IBD, thickening of the intestinal wall is associated with immune responses that can lead to inflammation and abdominal pain [71], and similar reactions have been reported in other species, including horses [72,73,74,75]. Given the potential pressure differences and closer interaction between host and non-host elements, the increased abundance of MGST1 in the pelvic flexure relative to the surrounding regions could indicate a need for more host cell protections.

Several genes demonstrated differential expression between the PF and segments of the ventral colon. C-reactive protein (CRP) was more abundant in the PF than in the preceding ventral colon. CRP is a biomarker of inflammation, involved in the activation of the complement system, and an essential part of host defense against pathogens [76]. GI microbiome differences have been associated with C-reactive protein levels, resulting in increased inflammation and changes to intestinal permeability with outcomes such as obesity and Crohn’s disease [77,78]. Therefore, the increased abundance of CRP observed at the PF could help explain the observed microbiome composition differences previously reported between the VC and DC [18]. A second gene, C-X-C motif chemokine ligand 6 (CXCL6), showed increased mRNA abundance in the PF versus the LVC. CXCL6 encodes a member of the CXC chemokine protein family. This protein is chemotactic for neutrophil granulocytes and antibacterial against certain gram-negative and gram-positive bacteria [79]. Interestingly, chemokines—including CXCL6—have been demonstrated to influence the abundance of gut microbe species and strains [80]. Interleukin 17 (IL-17)—implicated in inflammatory responses, neutrophil recruitment, and protection against extracellular bacterial pathogens—upregulates the expression of CXCL6 [81]. Higher levels of CXCL6 expression in the pelvic flexure could indicate targeting of specific microbes, which prevents their movement past the PF, resulting in the differentiation of the microbiota composition between the ventral and dorsal colons.

Our analysis also identified genes with differential expression between the PF and dorsal colon. Proteoglycan 3 (PRG3) was more highly expressed in the narrower and angled PF versus the broader and straighter dorsal colon. A recent study in mice examined differential gene expression associated with feeding a diet containing resistant potato starch (RPS). There was an observed increase in the abundance of *Citrobacter rodentium*—a pathogenic bacteria found in the mouse colon and shares 66.7% of encoded genes with *E. coli* [82]—in the distal colon of mice receiving RPS that correlated with, amongst other changes, a decrease in the expression of PRG3 [83]. The observed reduction in PRG3 expression in the dorsal colon relative to the PF could help explain differences in microbial content of the VC and DC by supporting environments that are more permissive to certain bacteria at specific locations. The PF must also resist compression to avoid collapse of the intestinal lumen, a function that expression of PRG3 supports [84]. A second gene with more abundant expression in the PF compared to the dorsal colon was MHC Class 1 heavy chain (MHCX1). MHCX1 is important in extracellular and intracellular pathogens’ signaling, binding, and immune responses [85]. Horses have high levels of variation in their MHC haplotypes [86,87], which could result in variations in the responses to bacteria and other microbes in the gut. Recent research has demonstrated that MHC heterozygosity promotes functional diversification of the microbiome, enhancing microbial network connectivity and enriching a variety of microbial functions that positively affect host fitness [88]. A final gene, Synaptotagmin 13 (or SYT13), was more abundant in the PF compared to the dorsal colon. SYT13 codes for a membrane trafficking protein actively involved in intracellular vesicle trafficking and exocytosis and, importantly, plays a role in modulating insulin secretion [89,90]. Insulin signaling has been demonstrated to shape gut community composition [90,91,92]. As a result, differential expression of SYT13 in the PF versus the dorsal colon could help determine microbiota composition before and after the pelvic flexure by influencing the regulation of insulin and nutrient transport.

## 5. Conclusions

The differences in gene expression between the ventral and dorsal colons in the equine hindgut relate to tissue function and could impact microbial composition. Previous results from Reed et al. demonstrated that differences exist between the microbial composition of the proximal and distal hindgut, pointing to the potential role of the pelvic flexure region in influencing these observed differences. Similarly, differences in epithelial tissue gene expression patterns were observed relative to the pelvic flexure and surrounding tissues. Differentially expressed genes play vital roles in immune function and the digestion and absorption of specific compounds, directly correlating to tissue-specific functions. One theory is that the pelvic flexure region acts not as a barrier to these microbes but as a “toll road”—the genes highly expressed in the pelvic flexure are directing who may pass through the pelvic flexure into the dorsal colon and who may not. A limitation of the current study is that the samples used for the analysis were not confirmed free of the serosal muscle layers. It is possible, therefore, that some of the gene expression results presented are influenced by contamination with RNA from these other layers. Future research could address this limitation and further analyze these differences by (a) expanding sample size and by sampling more locations in each hindgut region, (b) more narrowly focusing on the gene expression of specific genes that were found in this study, which could be closely directing these genetic, microbial and functional differences, and (c) introducing healthy versus unhealthy equine subjects to further the research community’s understanding of how health status may also affect gene expression and microbial communities in the equine hindgut.

## Figures and Tables

**Table 1:** Top 20 genes by expression value with restricted expression by tissue site.

Right Ventral Colon	Left Ventral Colon	Pelvic Flexure	Left Dorsal Colon	Right Dorsal Colon
Gene Symbol	Gene Symbol	Gene Symbol	Gene Symbol	Gene Symbol
*ADGRA1*	*FOXB1*	*SNORA19*	*LRRTM3*	*OR6B13*
*CFAP46*	*eca-mir545*	*NYX*	*SNORD108*	*PNLIP*
*CUZD1*	*IRS4*	*GPR119*	*CYP1A2*	*ANKRD1*
*CDHR1*	*eca-mir138-1*	*TKTL1*	*SLC24A1*	*SLC35F3*
*TMEM266*	*CDH8*	*TLE7*	*TGM5*	*SNORA74*
*DUT*	*eca-mir9074*	*DTHD1*	*DHRS2*	*GJD2*
*ACTC1*	*CSN1S1*	*eca-mir9077*	*ARSF*	*KLHL34*
*OR4F13*	*GABRG1*	*KLHDC7A*	*RS1*	*IL1RAPL1*
*MYH7*	*GJB4*	*SRARP*	*NCBP2L*	*PLAC1*
*TAF7L*	*HCRTR1*	*LRRD1*	*SNORA69*	*ENAM*
*RAB9B*	*ELOA*	*TAS2R3*	*TERB1*	*TMPRSS11A*
*C3H16orf86*	*PRSS55*	*OR2A75*	*MIR140*	*ADRA2C*
*AGBL4*	*eca-mir703*	*ASB10*	*C3H4orf17*	*CDCP2*
*ZMYND12*	*PON1*	*OAZ1*	*AMBN*	*eca-mir9061*
*POU3F1*	*DLX6*	*TRIM29*	*DMBX1*	*CITED4*
*OPRD1*	*CPA4*	*NTM*	*SCARNA1*	*TFAP2E*
*RPL11*	*PLK5*	*eca-mir1271b*	*XKR4*	*NR0B2*
*NPM2*	*GRIA4*	*PTPN5*	*SLC26A5*	*SLC30A2*
*GNRH1*	*OR7D20*	*SRRM4*	*CPA1*	*FAM131C*
*NPY2R*	*CCKBR*	*SNORA49*	*STRA8*	*CFAP74*

## Data Availability

The original data presented in this study are openly available in the Sequence Read Archive under Bioproject PRJNA631014.

## Supplementary Materials

The following supporting information can be downloaded at https://www.mdpi.com/article/10.3390/ani14162303/s1, Table S1: Sequencing results summary, Table S2: Mapping summary, Table S3: Tissue-restricted expression, Table S4: Differentially expressed genes RVC versus PF, Table S5: Differentially expressed genes LVC versus PF, Table S6: Differentially expressed genes LVC versus LDC, Table S7: Differentially expressed genes LDC versus PF, Table S8: Differentially expressed genes RDC versus PF, Table S9: Enriched GO categories RVC versus PF, Table S10: Enriched GO categories LVC versus PF, Table S11: Enriched GO categories LVC versus LDC, Table S12: Enriched GO categories LDC versus PF, Table S13: Enriched GO categories RDC versus PF.

## References

[1] Moore J.N., Melton T., Carter W.C., Wright A.L., Smith M.L. (2001). A new look at equine gastrointestinal anatomy, function, and selected intestinal displacements. AAEP Proc..

[2] Frape D. (2010). Equine Nutrition, and Feeding.

[3] Vermorel M., Martin-Rosset W. (1997). Concepts, scientific bases, structure and validation of the French horse net energy system (UFC). Livest. Prod. Sci..

[4] Julliand V., Grimm P. (2017). The impact of diet on the hindgut microbiome. JEVS.

[5] Bland S.D. (2016). Equine colic: A review of the equine hindgut and colic. Vet. Sci. Dev..

[6] Destrez A., Grimm P., Julliand V. (2019). Dietary-induced modulation of the hindgut microbiota is related to behavioral responses during stressful events in horses. Physiol. Behav..

[7] Lindenberg F., Krych L., Fielden J., Kot W., Frokiaer H., van Galen G., Nielsen D.S., Hansen A.K. (2019). Expression of immune regulatory genes correlates with the abundance of specific Clostridiales and Verrucomicrobia species in the equine ileum and cecum. Sci. Rep..

[8] Tavenner M.K., McDonnell S.M., Biddle A. (2020). Development of the equine hindgut microbiome in semi-feral and domestic conventionally managed foals. Anim. Microbiome.

[9] Park T., Cheong H., Yoon J., Kim A., Yun Y., Unno T. (2021). Comparison of the fecal microbiota of horses with intestinal disease and their healthy counterparts. Vet. Sci..

[10] Billman G.E. (2020). Homeostasis: The Underappreciated and Far Too Often Ignored Central Organizing Principle of Physiology. Front. Physiol..

[11] Sandler U., Tsitolovsky L. (2017). The S-Lagrangian and a theory of homeostasis in living systems. Phys. A Stat. Mech. Its Appl..

[12] Cooper S.J. (2008). From Claude Bernard to Walter Cannon. Emergence of the concept of homeostasis. Appetite.

[13] Brestoff J.R., Artis D. (2013). Commensal bacteria at the interface of host metabolism and the immune system. Nat. Immunol..

[14] de Fombelle A., Varloud M., Goachet A.G., Jacotot E., Philippeau C., Drogoul C., Julliand V. (2003). Characterization of the microbial and biochemical profile of the different segments of the digestive tract in horses given two distinct diets. Anim. Sci..

[15] Al Jassim R.A.M., Andrews F.M. (2009). The bacterial community of the horse gastrointestinal tract and its relation to fermentative acidosis, laminitis, colic, and stomach ulcers. Vet. Clin. N. Am. Equine Pract..

[16] Ericsson A.C., Johnson P.J., Lopes M.A., Perry S.C., Lanter H.R. (2016). A microbiological map of the healthy equine gastrointestinal tract. PLoS ONE.

[17] Su S., Zhao Y., Liu Z., Liu G., Du M., Wu J., Bai D., Li B., Bou G., Zhang X. (2020). Characterization and comparison of the bacterial microbiota in different gastrointestinal tract compartments of Mongolian horses. Microbiologyopen.

[18] Reed K.J., Kunz I.G.Z., Scare J.A., Nielsen M.K., Turk P.J., Coleman R.J., Coleman S.J. (2021). The pelvic flexure separates distinct microbial communities in the equine hindgut. Sci. Rep..

[19] Lopes M.A., Pfeiffer C.J. (2000). Functional morphology of the equine pelvic flexure and its role in disease. A review. Histol. Histopathol..

[20] Hintz H.F. (1975). Digestive physiology of the horse. J. S. Afr. Vet. Assoc..

[21] Santos A.S., Rodrigues M.A.M., Bessa R.J.B., Ferreira L.M., Martin-Rosset W. (2011). Understanding the equine cecum-colon ecosystem: Current knowledge and future perspectives. Animal.

[22] Hintz H.H., Schryver H.F., Stevens C.E. (1978). Digestion and absorption in the hindgut of nonruminant herbivores. J. Anim. Sci..

[23] Clauss M., Codron D., Hummel J. (2023). Equid nutritional physiology and behavior: An evolutionary perspective. J. Equine Vet. Sci..

[24] Henneke D.R., Potter G.D., Kreider J.L., Yeates B.F. (1983). Relationship between condition score, physical measurements and body fat percentage in mares. Equine Vet. J..

[25] Chomczynski P., Mackey K. (1995). Short technical reports. Modification of the TRI reagent procedure for isolation of RNA from polysaccharide- and proteoglycan-rich sources. BioTechniques.

[26] Rio D.C., Ares Jr M., Hannon G.J., Nilsen T.W. (2010). Purification of RNA using TRIzol (TRI reagent). Cold Spring Harbor Protocols.

[27] Galaxy Community (2022). The Galaxy platform for accessible, reproducible and collaborative biomedical analyses: 2022 update. Nucleic Acids Res..

[28] Babraham Bioinformatics: FastQC: A Quality Control Tool for High throughput Sequence Data. https://www.bioinformatics.babraham.ac.uk/projects/fastqc/.

[29] Ewels P., Magnusson M., Lundin S., Kaller M. (2016). MultiQC: Summarize analysis results for multiple tools and samples in a single report. Bioinformatics.

[30] Bolger A.M., Lohse M., Usadel B. (2014). Trimmomatic: A flexible trimmer for Illumina sequence data. Bioinformatics.

[31] Kim D., Paggi J.M., Park C., Bennet C., Salzberg S.L. (2019). Graph-based genome alignment and genotyping with HISAT2 and HISAT-genotype. Nat. Biotechnol..

[32] Cunningham F., Allen J.E., Allen J., Alvarez-Jarreta J., Amode M.R., Armean I.M., Austine-Orimoloye O., Azov A.G., Barnes I., Bennett R. (2022). Ensembl 2022. Nucleic Acids Res..

[33] Liao Y., Smyth G.K., Shi W. (2014). featureCounts: An efficient general purpose program for assigning sequence reads to genomic feature. Bioinformatics.

[34] Love M.I., Huber W., Anders S. (2014). Moderated estimation of fold change and dispersion for RNA-seq data with DESeq2. Genome Biol..

[35] Kendall M.G. (1938). A new measure of rank correlation. Biometrika.

[36] Brossart D.F., Laird V.C., Armstrong T.W. (2018). Interpreting Kendall’s Tau and Tau-U for Single-Case Experimental Designs. Cogent Psychol..

[37] calcTau: Implements Tissue Specificity Algorithm. https://rdrr.io/github/roonysgalbi/tispec/man/calcTau.html.

[38] Sherman B.T., Hao M., Qiu J., Jiao X., Baseler M.W., Lane H.C., Imamichi T., Chang W. (2022). DAVID: A web server for functional enrichment analysis and functional annotation of gene lists (2021 update). Nucleic Acids Res..

[39] Huang D.W., Sherman B.T., Lempicki R.A. (2009). Systematic and integrative analysis of large gene lists using DAVID bioinformatics resources. Nat. Protoc..

[40] Tuteja G., Kaestner K.H. (2007). SnapShot: Forkhead transcription factors I. Cell.

[41] van der Sluis M., Vincent A., Bouma J., Korteland-Van Male A., van Goudoever J.B., Renes I.B., Van Seuningen I. (2008). Forkhead box transcription factors Foxa1 and Foxa2 are important regulators of Muc2 mucin expression in intestinal epithelial cells. Biochem. Biophys. Res. Commun..

[42] Chen Z., Luo J., Li J., Kim G., Chen E.S., Xiao S., Snapper S.B., Bao B., An D., Blumberg R.S. (2021). Foxo1 controls gut homeostasis and commensalism by regulating mucus secretion. J. Exp. Med..

[43] Sanmartin-Salinas P., Guijarro L.G. (2018). Overexpression of IRS-4 Correlates with Procaspase 3 Levels in Tumoural Tissue of Patients with Colorectal Cancer. J. Oncol..

[44] Chatterjee I., Getselter D., Ghanayem N., Harari R., Davis L., Bel S., Elliot E. (2023). CHD8 regulates gut epithelial cell function and affects autism-related behaviors through the gut-brain axis. Transl. Psychiatry.

[45] Kopin A.S., Lee Y.M., McBride E.W., Beinborn M. (1992). Expression cloning and characterization of the canine parietal cell gastrin receptor. Proc. Natl. Acad. Sci. USA.

[46] Mjones P., Nordrum I.S., Sordal O., Sagatun L., Fossmark R., Sandvik A., Waldum H.L. (2018). Expression of the Cholecystokinin-B receptor in neoplastic gastric cells. Horm. Cancer.

[47] Cai X., Lytton J. (2004). Molecular cloning of a sixth member of the K^+^ -dependent Na^+^/Ca^2+^ exchanger gene family, NCKX6. J. Biochem..

[48] Lee S.I., Kim I.H. (2018). Difructose dianhydride improves intestinal calcium absorption, wound healing, and barrier function. Sci. Rep..

[49] Thelen K., Dressman J.B. (2010). Cytochrome P450-mediated metabolism in the human gut wall. J. Pharm. Pharmacol..

[50] Xie F., Ding X., Zhang Q.Y. (2016). An update on the role of intestinal cytochrome P450 enzymes in drug disposition. Acta Pharm. Sin. B..

[51] Journova L., Liskova B., Lnenickova K., Zemanova N., Anzenbacher P., Hermanova P., Hudcoivc T., Kozakova H., Anzenbacherova E. (2020). Presence of absence of microbiome modulates the response of mice organism to administered drug nabumetone. Physiol. Res..

[52] Wang S., Wen Q., Qin Y., Zia Q., Shen C., Song S. (2023). Gut microbiota and host cytochrome *P*450 characteristics in the pseudo germ-free model: Co-contributors to a diverse metabolic landscape. Gut Pathog..

[53] Cassidy A.J., van Steensel M.A.M., Steijlen P.M., van Geel M., van der Velden J., Morley S.M., Terrinoni A., Melino G., Candi E., Irwin McLean W.H. (2005). A homozygous missense mutation in TGM5 abolishes epidermal transglutaminase 5 activity and causes acral peeling skin syndrome. A J. Hum. Genet..

[54] Pigors M., Kiritsi D., Cobzaru C., Schwieger-Briel A., Suarez J., Faletra F., Aho H., Makela L., Kern J.S., Bruckner-Tuderman L. (2012). TGM5 mutations impact epidermal differentiation in acral peeling skin syndrome. J. Investig. Dermatol..

[55] Thomas M.L., Xu X., Norfleet A.M., Watson C.S. (1993). The presence of functional estrogen receptors in intestinal epithelial cells. Endocrinology.

[56] Looijer-van Langen M., Hotte N., Dieleman L.A., Albert E., Mulder C., Madsen K.L. (2011). Estrogen receptor-Œ≤ signaling modulates epithelial barrier function. Am. J. Physiol.-Gastrointest. Liver Physiol..

[57] Yang X., Guo Y., He J., Zhang F., Sun X., Yang S., Dong H. (2017). Estrogen and estrogen receptors in the modulation of gastrointestinal epithelial secretion. Oncotarget.

[58] Cima I., Corazza N., Dick B., Fuhrer A., Herren S., Jakob S., Ayuni E., Mueller C., Brunner T. (2004). Intestinal epithelial cells synthesize glucocorticoids and regulate T cell activation. J. Exp. Med..

[59] Tian N., Hu L., Lu Y., Tong L., Feng M., Liu Q., Li Y., Zhu Y., Wu L., Ji Y. (2021). TKT maintains intestinal ATP production and inhibits apoptosis-induced colitis. Cell Death Dis..

[60] Gil O.D., Zaanazzi G., Struyk A.F., Salzer J.L. (1998). Neurotrimin mediates bifunctional effects on neurite outgrowth via homophilic and heterophilic interactions. J. Neurosci..

[61] Krizsan-Agbas D., Pedchenko T., Smoth P.G. (2008). Neurotimin is an estrogen-regulated determinant of peripheral sympathetic innervation. J. Neurosci. Res..

[62] Hockley J.R.F., Taylor T.S., Callejo G., Wilbrey A.L., Gutteridge A., Bach K., Winchester W.J., Bulmer D.C., McMurray G., St John Smith E. (2019). Single-cell RNAseq reveals seven classes of colonic sensory neuron. Neurogastroenterology.

[63] Sano R., Nakajima T., Takahashi Y., Kubo R., Kobayashi M., Takahashi K., Takeshita H., Ogasawara K., Kominato Y. (2016). Epithelial Expression of Human ABO Blood Group Genes Is Dependent upon a Downstream Regulatory Element Functioning through an Epithelial Cell-specific Transcription Factor, Elf5. J. Biol. Chem..

[64] Makivuokko H., Lahtinen S.J., Wacklin P., Tuovinen E., Tenkanen H., Nikkila J., Bjorklund M., Aranko K., Ouwehand A.C., Matto J. (2012). Association between the ABO blood group and the human intestinal microbiota composition. BMC Microbiol..

[65] Wang Z., Potter C.S., Sundberg J.P., Hogenesch H. (2012). SHARPIN is a key regulator of immune and inflammatory responses. J. Cell. Mol. Med..

[66] Ikeda F., Deribe Y.L., Skanland S.S., Stieglitz B., Grabbe C., Franz-Wachtel M., van Wijk S.J., Goswami P., Nagy V., Terzic J. (2011). SHARPIN forms a linear ubiquitin ligase complex regulating NF-kB activity and apoptosis. Nature.

[67] Tokunaga F., Iwai K. (2012). Lubac, a novel ubiquitin ligase for linear ubiquitination, is crucial for inflammation and immune responses. Microbes Infect..

[68] Yu B., Wang F., Wang Y. (2022). Advances in the structural and physiological functions of SHARPIN. Front. Immunol..

[69] Gurung P., Sharma B., Kanneganti T.D. (2016). Distinct role of IL-1b in instigating disease in Sharpin^cpdm^ mice. Sci. Rep..

[70] Mu C., Yang Y., Luo Z., Guan L., Zhu W. (2016). The colonic microbiome and epithelial transcriptome are altered in rats fed a high-protein diet compared with a normal-protein diet. J. Nutr..

[71] Choy M.C., Visvanathan K., De Cruz P. (2017). An Overview of the Innate and Adaptive Immune System in Inflammatory Bowel Disease. Inflamm. Bowel Dis..

[72] Merck Manual, Veterinary Manual: Inflammatory Bowel Disease in Horses. https://www.merckvetmanual.com/digestive-system/miscellaneous-intestinal-diseases-in-horses/inflammatory-bowel-disease-in-horses.

[73] Kalck K.A. (2009). Inflammatory bowel disease in horses. Vet. Clin. N. Am. Equine Pract..

[74] Vitale V. (2021). Inflammatory bowel diseases in horses: What do we know?. Equine Vet. Educ..

[75] Olofsson K.M. (2016). Immunopathological Aspects of Equine Inflammatory Bowel Disease. Ph.D. Thesis.

[76] Al Bander Z., Dekker Nitert M., Mousa A., Naderpoor N. (2020). The gut microbiota and inflammation: And overview. Int. J. Environ. Res. Public Health.

[77] Verdam F.J., Fuentes S., de Jonge C., Zoetendal E.G., Erbil R., Greve J.W., Buurman W.A., de Vos W.M., Rensen S.S. (2013). Human intestinal microbiota composition is associated with local and systemic inflammation in obesity. Obes. Biol. Integr. Physiol..

[78] Karl J.P., Margolis L.M., Madslien E.H., Murphy N.E., Castellani J.W., Gundersen Y., Hoke A.V., Levangie M.W., Kumar R., Chakraborty N. (2017). Changes in intestinal microbiota composition and metabolism coincide with increased intestinal permeability in young adults under prolonged physiological stress. Am. J. Physiol. Gastrointest Liver Physiol..

[79] Linge H.M., Collin M., Nordenfelt P., Morgelin M., Malmsten M., Egesten A. (2008). The human CXC chemokine granulocyte chemotactic protein 2 (GCP-2)/CXCL6 possesses membrane-disrupting properties and is antibacterial. Antimicrob. Agents Chemother..

[80] Lloyd-Price J., Arze C., Ananthakrishan A.N., Schirmer M., Avila-Pacheco J., Poon T.W., Andrews E., Ajami N.J., Bonham K.S., Brislawn C.J. (2019). Multi-omics of the gut microbial ecosystem in inflammatory bowel diseases. Nature.

[81] Vitiello G.A., Miller G. (2020). Targeting the interleukin-17 immune axis for cancer immunotherapy. J. Exp. Med..

[82] Collins J.W., Keeney K.M., Crepin V.F., Rathinam V.A.K., Fitzgerald K.A., Finlay B.B., Frankel G. (2014). *Citrobacter rodentium*: Infection, inflammation, and the microbiota. Nat. Rev. Microbiol..

[83] Smith A.D., Chen C., Cheung L., Dawson H.D. (2023). Raw potato starch alters the microbiome, colon and cecal gene expression, and resistance to *Citrobacter rodentium* infection in mice fed a Western diet. Front. Nutr..

[84] NIH National Library of Medicine, National Center for Biotechnology Information: PRG3 Proteoglycan 3, Pro-Eosinophil Major Basic Protein 2 [Homo Sapiens (Human)]. https://www.ncbi.nlm.nih.gov/gene/10394.

[85] Janeway C.A., Travers P., Walport M., Shlomchik M.J. (2001). The major histocompatibility complex and its functions. Immunobiology: The Immune System in Health and Disease.

[86] Sadeghi R., Moradi-Shahrbabak M., Miraei Ashtiani S.R., Miller D.C., Antczak D.F. (2017). MHC haplotype diversity in Persian Arabian horses determined using polymorphic microsatellites. Immunogenetics.

[87] Plasil M., Oppelt J., Klumplerova M., Bubenikova J., Vychodilova L., Janova E., Stejskalova K., Futas J., Knoll A., Leblond A. (2023). Newly identified variability of the antigen binding site coding sequences of the equine major histocompatibility complex class I and class II genes. HLA.

[88] Khan M.A.W., Stephens W.Z., Mohammed A.D., Round J.L., Kubinak J.L. (2019). Does MHC heterozygosity influence microbiota form and function?. PLoS ONE.

[89] Bakhti M., Bastidas-Ponce A., Tritschler S., Czarnecki O., Tarquis-Medina M., Nedvedova E., Kaki J., Willmann S.J., Scheibner K., Cota P. (2022). Synaptotagmin-13 orchestrates pancreatic endocrine cell egression and islet morphogenesis. Nat. Commun..

[90] Ofori J.K., Karagiannopoulos A., Barghouth M., Nagao M., Andersson M.E., Salunkhe V.A., Zhang E., Wendt A., Eliasson L. (2022). The highly expressed calcium-insensitive synaptotagmin-11 and synaptotagmin-13 modulate insulin secretion. Acta Physiol..

[91] Hu P.J. (2021). Microbiome: Insulin signaling shapes gut community composition. Curr. Biol..

[92] Rahman M.S., Hossain K.S., Das S., Kundu S., Adegoke E.O., Rahman M.A., Hannan M.A., Uddin M.J., Pang M.G. (2021). Role of Insulin in Health and Disease: An Update. Int. J. Mol. Sci..

